# Clinical and radiographic evaluation of applying 1% metformin biofilm with plasma rich in growth factor (PRGF) for treatment of two-wall intrabony periodontal defects: A randomized clinical trial 

**DOI:** 10.15171/joddd.2019.008

**Published:** 2019-04-24

**Authors:** Shabnam Khalifehzadeh, Sina Haghanifar, Niloofar Jenabian, Sohrab Kazemi, Mahmoud Hajiahmadi

**Affiliations:** ^1^Student Research Committee, Babol University of Medical Sciences, Babol, Iran; ^2^Oral Health Research Center, Health Research Institute, Babol University of Medical Sciences, Babol, Iran; ^3^Dental Materials Research Center, Health Research Institute, Babol University of Medical Sciences, Babol, Iran; ^4^Cellular and Molecular Biology Research Center Institute, Babol University of Medical Sciences, Babol, Iran; ^5^Department Biostatistics and Epidemiology, Babol University of Medical Sciences, Babol, Iran

**Keywords:** Intrabony two-wall periodontal defects, Metformin 1%, plasma rich in growth factor, regeneration

## Abstract

***Background***. The ultimate aim of periodontal treatment is to regenerate periodontium and regenerative treatment after that. The aim of this study was to evaluate the effect of PRGF with 1% metformin biofilm in the treatment of two-wall intrabony periodontal defects.

***Methods***. In this clinical trial, 8 patients with moderate chronic periodontitis and two-wall intrabony defect were selected. The defects were assigned to 4 groups: debridement, 1% metformin, PRGF, PRGF and metformin. The parameters of vertical probing depth, vertical clinical attachment level and gingival index were measured at baseline, immediately before surgery, and 3 and 6 months after surgery. In addition, the radiographic changes were evaluated with digital subtraction radiography before and 6 months after surgery. Analysis of the results was performed with repeated measurements, Friedman test and chisquared test.

***Results***. All the groups exhibited improvements in all the clinical parameters after 6 months. Inter-group comparison of GI, CAL and PPD parameters revealed no statistically significant differences. Radiographic changes in the group of 1% metformin with PRGF revealed statistically significant differences compared with other groups; however, there were no statistically significant differences in other groups.

***Conclusion***. Application of PRGF with 1% metformin in intrabony two-wall periodontal defects was effective in improving the clinical parameters but this effect revealed no difference compared with other groups; however, in terms of radiographic changes significant improvements were noted

## Introduction

Angular intrabony defects are signs of periodontal disease progression. To date, many techniques, including resective and regenerative procedures, have been applied for the treatment of periodontal intrabony defects. Resective techniques remove granulation tissues but do not regenerate the periodontium. The goal of periodontal treatment is to regenerate the damaged periodontal structures.^1^ Many studies have shown that the growth factors play an important role in periodontal healing and regeneration.^2,3^ Biologically active endogenous proteins offer a new approach to tissue regeneration.^3^ In 1999, Anitua‏ explained a new technique for preparing plasma rich in growth factors (PRGF). This 100% autologous preparation is enriched with biological mediators that accelerate regeneration of both hard and soft tissues.^2^ PRGF contains a high concentration of a platelet‑derived growth factor, insulin‑like growth factor, and fibroblast growth factor and due to the lack of leukocyte has a minimum concentration of proinflammatory interleukins.^4-6^

Metformin (MF) is a biguanide that is one of the most common oral hypoglycemic drugs used for the treatment of type II diabetes mellitus. The action of MF on the development of osteoblast-like cell lines was checked out for the first time by Cortizo et al, who showed a direct osteogenic effect of MF on osteoblasts in culture. The effect of the systemic administration of MF on alveolar bone resorption and the ratio of the RANKL/OPG in rats offered to experimental periapical lesions showed a decrease in the number of RANKL-positive cells and an increase in OPG-positive cells. The periapical bone loss area in the MF-treated group significantly decreased.^7,8^

The existing literature is scarce‏ for the application of a combination of PRGF (i.e., not PRP) with metformin in the intrabony periodontal defects; hence, the current research was conducted to evaluate the effect of combined PRGF and 1% metformin on the treatment of two-wall intrabony periodontal defects.

## Methods

### Randomization and Blindness

This study was designed as a randomized, double‑blinded (patient, clinician) study. Eight patients referring to the Periodontology Department of Babol University of Medical Sciences with moderate chronic periodontitis and two-wall intrabony periodontal defects were included in this study. The defects were assigned randomly to 4 groups for treatment with PRGF, MF, MF and PRGF or control groups and 6 defects were placed in each group. The clinician responsible for performing the interventions was unaware of the assignment codes. Further measurements of the periodontal indices were performed by another clinician who was blinded to the study groups. A maxillofacial radiologist blindly reported the radiographic changes. This study was approved by the Ethics Committee of the university under the code MUBABOL.REC.1396.49. It was also registered in the WHO clinical trial registry, branch of the Islamic Republic of Iran under the code IRCT: 20100427003813N6.

**Inclusion criteria:** 1) a minimum patient age of 18 years old; 2) chronic periodontal disease with two-wall intrabony defects; 3) similar plaque index; 4) the ability to maintain proper oral hygiene; and 5) signing an informed consent form.
**Exclusion criteria were defined as**: 1) pregnancy; 2) coagulation problems; 3) use of medications interfering with platelet function; 4) use of drugs interfering with wound healing (e.g. corticosteroids); 5) any local or systemic disease preventing periodontal surgery; 6) any known allergies to the materials used and any contraindication for periodontal surgery; 7) any active disease (e.g. HIV); and 8) lack of interest in accepting a periodontal surgery and compliance during follow-ups.

### Study Protocol

All the enrolled patients signed a written informed consent form. Oral hygiene instruction, scaling and root planing (SRP) were provided to all the patients to achieve a satisfactory level of biofilm before surgery. Occlusion was corrected if needed. All the surgical procedures were performed by the same person: intrasulcular incision with mucoperiosteal flap elevation, debridement of granulation tissue, subgingival SRP, and rinsing with normal saline solution. Then, the patients were assigned to the groups based on the predefined random allocation.

**Figure 1:**
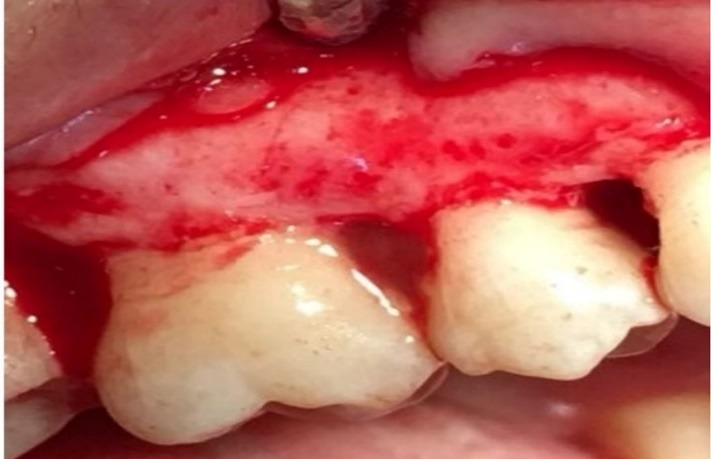
Defect zone containing metformin.

Group 1 then received debridement only.
Group 2 underwent debridement and 1% MF was placed in the defects (Figure 1).

**Figure 2:**
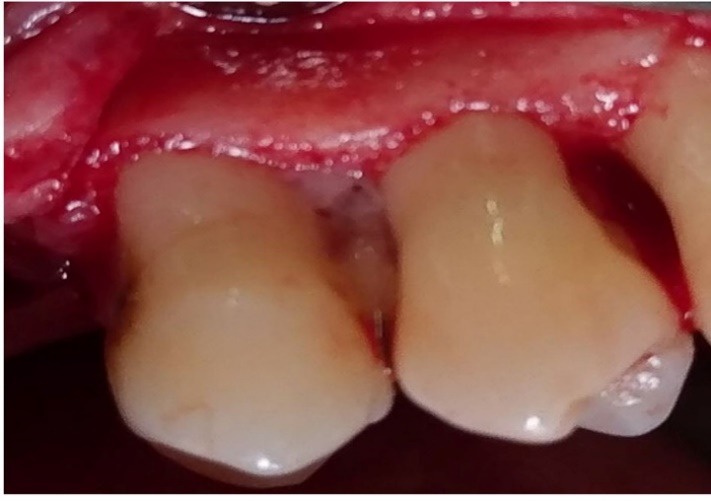
Defect zone containing PRGF.

**Figure 3:**
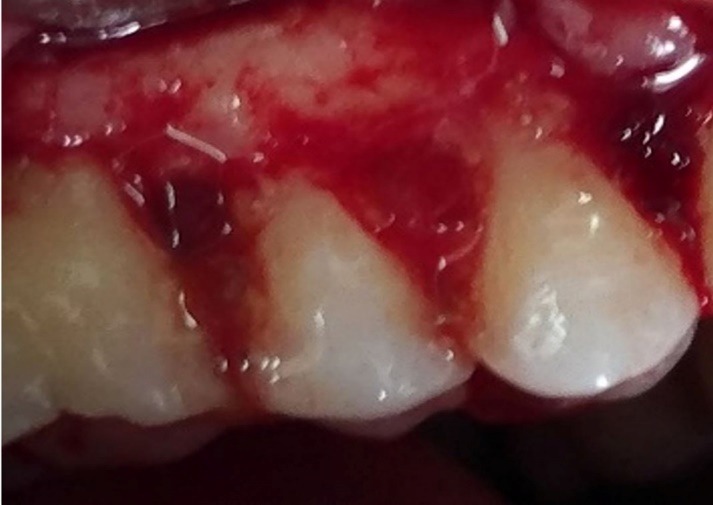
Defect zone containing metformin and PRGF.

Group 3 underwent debridement and then the lesions were fully filled with PRGF gel (Figure 2); and in group 4 after debridement, first 1% MF biofilm was placed in the PRGF gel to intake gel and then the biofilm was packed into the lesions and PRGF gel was placed in the lesions over the biofilm (Figure 3).

Then, the flap was sutured in place with silk 3-0 sutures, and the patients were discharged with antibiotic prescription (amoxicillin, 500 mg, TID), the directions for oral hygiene (0.12% chlorhexidine, 5 mL, BID) and with the recalls schedule.

### Clinical Parameters

Recording of the baseline measurements was performed by‏ a graduated periodontal probe.

The clinical parameters were as follows: VCAL (the distance from the CEJ [cementoenamel junction] to the depth of pocket); VPD (the distance from the free gingival margin to the gingival sulcus); and GI (Silness and Loe index). The parameters were measured at the beginning of the study (T0), at the time of surgery (T1), 3 months later (T2) and 6 months after the surgery (T3).

### Preparation of PRGF

Blood harvesting was performed a few minutes before surgery (10 mL). Each 4.5 mL of collected blood‏ was mixed in sterilized tubes with 0.5 mL of 3.8% sodium‏ citrate as an anticoagulant. The final preparation‏ was centrifuged with a single-speed device at 460 g‏ for 8 minutes (PRGF‑Endoret System IV Biotechnology‏ Institute, Vitoria, Spain). Then,‏ plasma was divided into three parts, including PRGF,‏ the plasma poor in GF, and plasma moderate in GF.‏ The PRGF was the buffy coat just above the red‏ blood cells within the tube, and it was collected with a 100-μL micropipette and each‏ 1 mL of it was mixed with 50 μL of 10% calcium chloride.‏ This resulted in a jelly‏ product saved in a sterilized‏ concealed glass container until surgery began.^9^

### Preparation of 1% Metformin Biofilm

For the preparation of biofilm, 95 mL of distilled water were transferred into a 500-mL Erlenmeyer container which was placed on a hot plate shaker. Then the required carbopol was added to water and heated; after that, the methyl paraben and propyl paraben were solved in 95% alcohol and the solution was added to the beaker, too.

Finally, the required amount of glycerine was added to the sample. Then 0.5 gram of metformin was added to this base gel per 100 mL of gel for preparing the biofilm.^10^

### Radiologic Assessments

The first radiograph was taken by the PSP Digital Sensor Size 2, using the parallel technique (PCT, Soredex;‏ Helsinki, Finland). Bite registration was performed by‏ using acrylic‏ resin (Duralay, Reliance, Worth, IL, USA)‏ that was at first recorded during the radiography to‏ ensure the same occlusion during the recall radiography.‏ Hence, the second radiography was taken 6 months‏ later with the same kV, mA, exposure‏ time, and the same recorded occlusion. Images were recorded as‏ DICOM series and processed by using Digora for Windows, Version 2.5 (PCT, Soredex; Helsinki, Finland). The digital subtraction of pre‑ and post‑treatment images was carried out by Photoshop CS6 software (Adobe Systems, California, USA). Serial digital images were produced in a parallel manner so that they could be superimposed, and a combination of two images could be seen on the screen. When the two images are recorded from the same object and the image intensities of corresponding pixels are subtracted, the difference will create a single image. This technique is called digital subtraction radiography (DSR).^11,12^ The findings are interpreted as osteogenesis if radiopacity is observed and as resorption if difference of density is seen as radiolucency; otherwise, it would be reported as “no change.”

### Statistical Analysis

Quantitative data were recorded as means ± standard deviations (medians). To compare the means, a parametric test‏ (repeated measurement) and for qualitative variables non-parametric tests‏ (Friedman, chi-squared) were used. Data were analyzed using SPSS 22 at a significance level of P<0.05.

## Results

In this clinical trial, 8 patients were finally enrolled, and 24 sites were treated by either open flap debridement/metformin/PRGF/metformin + PRGF. Five patients were female and‏ 3 were male; their age ranged from 25 to 45 years with a mean age of 35 years.

### Radiologic Findings

Interpretations of radiographs are displayed in Table 1. Chi-squared test showed statistically significant differences between MF and PRGF groups (P=0.031, χ^2^(2) = 13.855).‏

**Table 1:** Radiographic changes by treatment and intervention

**Results**	**Control**	MF*	PRGF**	**PRGF and MF**
**Radiolucency**	2	0	0	0
**No changes**	4	4	5	2
**Radiopacity**	0	2	1	4

*Metformin, **Plasma rich in growth factor

**Figure 4:**
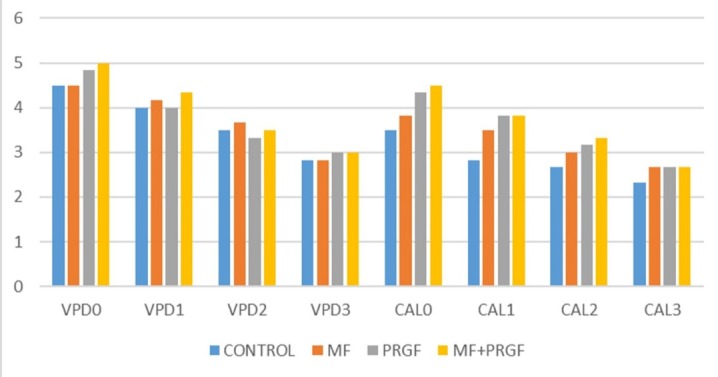
Clinical parameters by treatment group.

Data regarding clinical parameters of patients are displayed in Table 2 and Figure 4. Comparison of mean HPD, VPD and VCAL shoed differences between the four study groups from T0 to T4 by repeated measurement test. The clinical parameters did not improve significantly (First error was 0.05) (VCAL: P=0.088, VPD: P=0.945). However, there were significant differences between the groups from the beginning to the end of treatment (P<0.001).

**Table 2:** P-values in comparisons between the four treatment groups

	**P-value**			
**Parameter**	**T0**	**T1**	**T2**	**T3**
GI*	0.261	0.104	0.271	0.101

*Gingival index

Comparison of mean GI between the four study groups from T0 to T4 by chi-squared and Friedman tests showed that the clinical parameters did not improve significantly (first error was 0.05) (Table 3).

**Table 3:** P-values in comparisons between the treatment groups

	**P-value**			
**Parameters**	**Control**	MF*	PRGF**	**PRGF+MF**
GI***	0.001	0.001	0.001	0.001

*Metformin, **Plasma rich in growth factor, ***Gingival index

## Discussion

The present study was conducted on patients with moderate chronic periodontitis with two-wall intrabony periodontal defects. The study groups consisted of open flap debridement/metformin/PRGF/metformin + PRGF.

This results showed that treatment of two-walls intrabony defect with PRGF, MF or PRGF + MF did not significantly improve clinical parameters (VPD, VCAL and GI) in comparison with the control group; however, data improved significantly in each group from the beginning to the end of the study.

Radiographic changes showed statistically significant differences in PRGF + MF group 6 months after surgery.

Advantages of using PRGF instead of other PRP systems (the convenience of performing the technique, applicable in the office and in the hospital environment, need for a simpler and less expensive equipment, minimizing patient discomfort when taking blood because of the need for a small amount of blood, reducing the risk of infection and the minimum time required to prepare)^9^ were the reasons for using PRGF and metformin in two-wall intrabony periodontal defects.

Pradeep et al (2012) reviewed the efficacy of varying concentrations of subgingivally delivered metformin in the treatment of chronic periodontitis. They used 0.5%, 1% or 1.5% MF gel or placebo gel. The results showed that local delivery of MF into the periodontal pocket resulted in a significant increase in PD reduction, CAL gain and intrabony defect depth reduction compared to placebo as adjuncts to SRP 6 months after treatment.^13^ In the present study also 1% metformin was used. According to Haghpanah et al study ‏)2015), biofilm has the capacity for prolonged release of effective substances at the site of the lesion.^10^ This benefit led to use of this pharmaceutical form instead of metformin gel in this study.

Pradeep et al (2015) investigated the effect of platelet-rich fibrin with 1% metformin for the treatment of intrabony defects in chronic periodontitis. The results showed that PRF + 1% MF group exhibited greater improvements in clinical parameters, with greater percentage of radiographic defect depth reduction compared to MF, PRF, or debridement alone in the treatment of intrabony defects.^14^ In the present study also, use of MF with PRGF was effective in improving the level of the clinical parameters;‏ however, it was not significant compared with other groups. This difference in outcome is probably due to a smaller sample size in the present study.

In terms of radiographic changes, significant improvements were shown in MF + PRGF group; however, most of the defects in the MF and PRGF groups had no radiographic changes and two defects in the control group showed radiolucency, which might be the reason for the significance of radiographic changes unlike the clinical parameters.

In‏ many studies evaluating the effect of PRGF on‏ bone regeneration, PRGF was used in combination with‏ autografts. This results in transportation of vital cells in‏ the defect^2,15,16^ and tissue regeneration only occurs in‏ sites with vital cells, affected by signaling molecules‏ such as growth factors.^17^ Unlike large intrabony defects in previous studies,^2,15,16^ smaller periodontal defects were treated in this study. It was assumed that the vital‏ cells surrounding the intrabony lesion provide sufficient‏ cells for the growth factors in PRGF; therefore, in the present study no‏ additional bone materials or autografts were used. For‏ future studies, it is recommended to evaluate the effect‏ of PRGF in combination with autografts.

## Conclusion

Use of PRGF with 1% metformin in two-wall intrabony periodontal defects was effective in improving clinical parameters but this effect did not differ from other groups. However, in term of radiographic changes there were no significant improvements.

## Conflict of Interests

The authors declare that they have no conflict of interests, real or perceived, financial or non-financial in

## Authors’ contributions

The study was planned by NJ and SH. Data collection was carried out by SHKH; statistical analyses and interpretation of data were carried out by MH; The manuscript was prepared by SHKH and edited by NJ and SH; All the authors critically revised the manuscript for intellectual content. All the authors have read and approved the final manuscript for submission.

## Acknowledgments

This study was supported by the Research Council of Babol University of Medical Sciences. The authors would like to thank the Council for assisting in carrying out this study.

## Funding

This study was a part of research project (Grant No. 964449) which was supported and funded by Babol University of Medical Sciences.

## Ethics approval

This study was approved by the ethical committee of university(MUBABOL.REC.1396.49).
